# Longitudinal Study of Reproductive Performance of Female Cattle Produced by Somatic Cell Nuclear Transfer

**DOI:** 10.1371/journal.pone.0084283

**Published:** 2013-12-31

**Authors:** Irina A. Polejaeva, Diane M. Broek, Shawn C. Walker, Wenli Zhou, Mark Walton, Abby D. Benninghoff, David C. Faber

**Affiliations:** 1 Department of Animal, Dairy and Veterinary Sciences, Utah State University, Logan, Utah, United States of America; 2 ViaGen, L.C., Cedar Park, Texas, United States of America; 3 Recombinetics, Inc., St. Paul, Minnesota, United States of America; 4 School of Veterinary Medicine, Utah State University, Logan, Utah, United States of America; 5 Trans Ova Genetics, L.C., Sioux Center, Iowa, United States of America; University of Connecticut, United States of America

## Abstract

The objective of this study was to determine whether or not reproductive performance in cattle produced by somatic cell nuclear transfer (SCNT) is significantly different from that of their genetic donors. To address this question, we directed two longitudinal studies using different embryo production procedures: (1) superovulation followed by artificial insemination (AI) and embryo collection and (2) ultrasound-guided ovum pick-up followed by *in vitro* fertilization (OPU-IVF). Collectively, these two studies represent the largest data set available for any species on the reproductive performance of female clones and their genetic donors as measured by their embryo production outcomes in commercial embryo production program. The large-scale study described herein was conducted over a six-year period of time and provides a unique comparison of 96 clones to the 40 corresponding genetic donors. To our knowledge, this is the first longitudinal study on the reproductive performance of cattle clones using OPU-IVF. With nearly 2,000 reproductive procedures performed and more than 9,200 transferable embryos produced, our observations show that the reproductive performance of cattle produced by SCNT is not different compared to their genetic donors for the production of transferable embryos after either AI followed by embryo collection (*P = *0.77) or OPU-IVF (*P = *0.97). These data are in agreement with previous reports showing that the reproductive capabilities of cloned cattle are equal to that of conventionally produced cattle. In conclusion, results of this longitudinal study once again demonstrate that cloning technology, in combination with superovulation, AI and embryo collection or OPU-IVF, provides a valuable tool for faster dissemination of superior maternal genetics.

## Introduction

Somatic cell nuclear transfer (SCNT), or cloning, is one of the assisted reproductive technologies currently used in agriculture. Assisted reproductive technologies (ART) include artificial insemination (AI), multiple ovulation and embryo transfer (MOET), *in vitro* fertilization (IVF), semen sexing and SCNT. Commercial applications of SCNT in agriculture are presently limited to the production of animals of high genetic merit or the production of the most elite show cattle [1]. Production of bovine clones of elite bulls [2]–[4], cows with high milk performance [5], [6] and rare or endangered breeds to maintain genetic diversity have also been reported [7], [8]. One of the benefits of ART is to increase the presence of desirable characteristics (increased feed efficiency, reduced waste, disease resistance [*e.g.,* mastitis]) in production herds. Previously, population outliers (especially maternal lines) had insignificant impact on the population mean. Cloning has the potential to impact animal breeding in a fundamental way by amplifying the impact of unique genotypes in a population and enhancing the traits of interest [9]. Cloning can specifically leverage superior female genetics to a much greater extent by creating multiple copies of an elite individual followed by the subsequent use of a variety of assisted reproductive technologies (*e.g.,* AI, MOET, IVF) that would allow faster dissemination of unique genetic traits.

In order to capitalize on this process of genetic trait propagation, it is essential to evaluate the reproductive performance of animals generated by SCNT. Several studies have indicated that cloned animals have normal reproductive characteristics [10]–[15]. The reproductive performance of clones, including the production of semen by males or oocytes by females, embryo development, conception rates and gestation length, were reported to be normal. No difference was reported in the litter size of pigs, the birth weight or the peri- and pre-weaning mortality rates between the mating of clones and conventionally produced pigs [14], [15]. Furthermore, the progeny of animal clones exhibited normal phenotypic characteristics. Their growth, health and hematological parameters were comparable to the age-matched control animals [3]. In most of these studies, the reproductive performance of clones was assessed after AI or natural mating. Additionally, Panarace and co-workers evaluated the reproductive performance of cloned heifers and cows in an embryo transfer program and found no difference in the number of viable embryos per embryo collection between the clones and their controls [10]. Nevertheless, no longitudinal study on the reproductive performance of cattle clones is currently published. Additionally, to our knowledge, no data have been reported on the reproductive performance of clones using ultrasound-guided transvaginal oocyte retrieval (Ovum pick-up (OPU)) followed by *in vitro* fertilization (IVF). Since the late 1980’s, OPU-IVF has become a well-established embryo production technique [16]. International Embryo Transfer Society (IETS) statistics indicate that nearly 374,000 *in vitro*–produced embryos were transferred worldwide in 2011, an increase of 10% from 2010 (340,000) [17]. Cloning used in combination with OPU-IVF could substantially enhance the dissemination of superior maternal genetics. Therefore, it is important to assess reproductive performance of clones using this method.

The objective of this study was to determine if the reproductive performance in cattle produced by SCNT is significantly different from that of their genetic donors. To address the question we directed two longitudinal studies using either (1) superovulation and AI followed by embryo collection, or (2) ultrasound-guided OPU followed by IVF (OPU-IVF).

## Materials and Methods

### Animals

All *in vivo* procedures described in this manuscript were conducted strictly for agricultural purposes. The study and analysis were designed and conducted retrospectively, using only existing data. All animals were housed in donor housing at Trans Ova Genetics embryo transfer facilities in Sioux Center, Iowa. The housing facilities include dry lots with grass exercise areas and sheltered housing for inclement weather. The female cattle were fed free choice high quality grass hay and a daily total mixed ration of mixed grains and chelates minerals based on body condition. Veterinary oversight was provided on all donors. Trained donor herdsmen provided daily care and feeding. They also monitored cattle health status and observed estrus multiple times per day. Donor palpations, superovulation, OPU and embryo collection procedures were performed by trained, accredited veterinarians. Lidocaine was used for epidural anesthesia prior to OPU or embryo collection that is standard agricultural practice.

Clones (heifers and cows) used in this study were generated from adult fibroblast cells using somatic cell nuclear transfer as previously described [18]. Adult fibroblast cells were derived from a skin biopsy sample and prepared for cloning as described elsewhere [19]. Skin biopsy samples were collected from either the ear or the tail of elite genetic donors. In total, 96 clones and 40 corresponding genetic donors were used in the two longitudinal studies described below (Table 1). Embryo donor numbers were not static as the animals were actively moving in and out of the program. Some embryo donors were used for only one embryo collection or OPU-IVF procedure, whereas others underwent several collections. The majority of transferable embryos generated in these longitudinal studies were either frozen for future use or shipped for embryo transfer to a client location. Some of the embryos were transferred into recipients immediately after embryo quality assessment. However, the recipients were typically transported to client-managed locations. Due to our inability to monitor pregnancy and calving outcomes, the number of transferable embryos was used as the end point for the reproductive performance assessment. Embryo viability was assessed using the IETS scoring system, which is the standard reference for embryos that are exported and imported internationally [20]. All data were collected over a six-year period of time from January 2007 to January 2013 in a commercial setting at Trans Ova Genetics, Sioux Center, Iowa.

**Table 1:** Number of genetic donors and clones used in longitudinal studies.

AI with embryo collection		Ovum Pickup with IVF	
Genetic Donor ID	Number of Clones	Genetic Donor ID	Number of Clones
ID-123	1	ID-123	2
ID-127	1	ID-142	2
ID-142	4	ID-168	1
ID-168	1	ID-218	2
ID-201	3	ID-273	1
ID-209	3	ID-321	1
ID-257	3	ID-408	9
ID-273	1	ID-434	2
ID-320	3	ID-446	1
ID-355	1	ID-557	10
ID-378	2	ID-559	2
ID-408	8	ID-615	1
ID-446	2	ID-622	2
ID-450	6	ID-705	1
ID-464	2	ID-740	1
ID-470	2	ID-753	4
ID-501	1	ID-754	2
ID-516	2	ID-787	2
ID-557	11	ID-794	5
ID-559	3	ID-818	2
ID-615	1	ID-836	2
ID-705	1	ID-836	2
ID-740	1	ID-877	2
ID-753	2	ID-918	1
ID-754	2	ID-962	1
ID-766	2		
ID-787	3		
ID-794	5		
ID-836	1		
ID-836	2		
ID-846	1		
ID-889	2		
ID-918	1		
ID-962	1		
***Total number per group***			
**34**	**85**	**25**	**61**

### Longitudinal Study 1

The first study focused on the evaluation of the embryo production outcomes between clones and their genetic donors after superovulation followed by AI and embryo collection. Two parameters were used to assess breeding outcomes: the number of flushed embryos and the number of transferable embryos.

#### Embryo donor synchronization and superovulation

All embryo donors were open cycling heifers that were at least 12 to 14 months of age or adult cows that were approximately 60 days post calving. Embryo donors were synchronized with one or two injections of prostaglandin F2 alpha (Estrumate, Schering Plough) and observed for estrus or they were observed on a natural estrus. Superovulation treatments began from 8 to 12 days post estrus (Day 0 = estrus). Embryo donors received twice daily injections of FSH (Folltropin, Bioniche Animal Health or Pluset, Minitube; both contain 20 IU of FSH per ml) for four days in a decreasing dose regime. The injections were approximately 12 hours apart. Doses depended on the parity, weight, age and breed of the donor. Heifer donors received a total of 25 to 35 mg of FSH and adult donors received a total of 30 to 45 mg of FSH in their superovulation regimes. On the fourth day of the FSH regime, two injections of prostaglandin PGF2alpha (Lutalyse – Pfizer or Estrumate, Schering Plough) were administered. All injections were intramuscular. Embryo donors were observed in estrus approximately 36 to 48 hours following the first prostaglandin injection at the end of the 4-day superovulation regime.

#### Artificial insemination and embryo collection

Embryo donors were artificially inseminated beginning at 12 hours post first standing estrus and again 12 hours later. Embryos were collected seven days post insemination using the interrupted-syringe method [21]. A 16 or 18–20 gauge sterile catheter was used to perform the embryo collection for heifers and cows, respectively. Approximately 300 to 500 ml of flush medium (Bioniche, Animal Health) was processed through each of the two uterine horns to ensure that they were adequately flushed. The entire collection of flush media was processed through an Em Con filter with the excess fluid drained, leaving approximately 50 ml of flush media containing the embryos that was moved to the laboratory for searching, grading and classifying. Embryo viability was assessed using morphological criteria as outlined in the IETS embryo scoring system, which is based on embryo developmental stage and cytoplasmic characteristics [20]. Multiple embryo collection attempts were usually performed for each genetic donor or clone, and the average number of flushed embryos and transferable embryos was calculated on a per animal bases and used for comparison. If there were multiple clones from one genetic donor, the average number of flushed embryos or transferable embryos was calculated from each of the clones.

### Longitudinal Study 2

The second study compared the embryo production outcomes between clones and their genetic donors after ovum pick-up followed by *in vitro* fertilization (OPU-IVF). To stimulate follicular recruitment for the OPU procedure, oocyte donors received a single 2 ml GnRH injection, and then 36–48 hours later four FSH injections were given 12 hours apart (10–14 ml total per donor or 120–160 IU each). The OPU procedures were performed 24–36 hours after the last FSH injection as previously described [22], but with different probes. Probes with frequencies that oscillate between 5.0 and 7.5 MHz were used. The vacuum pressure used for aspiration of the cumulus oocyte complexes (COCs) varied from 65 mm Hg to 75 mm Hg depending on the combination of needle diameter and length of interconnector tubing to the container. In order to guarantee the number and desired quality of the COCs, a flow of 20–25 ml per minute in the collection system was used [23]. The COCs from each heifer or cow were submitted to the IVF lab, and the oocytes were rinsed, harvested, and graded according to the layers of cumulus cells and the appearance of the cytoplasm of the oocyte. The resulting oocytes were cultured in oocyte maturation medium as describe elsewhere [22] for 20–24 hours. The oocytes were then inseminated with reverse sorted semen, frozen sorted semen or non-sorted semen in fertilization medium. Typically semen sex sorting procedure is performed before cryopreservation. When sex sorting occurs after the semen has been previously frozen the process is called reverse semen sorting. Reverse sorting of frozen semen was performed at TransOva Genetics using a flow cytometer (Moflo SX, Dakocytomation, Ft. Collins, Colorado, USA). Post-thaw sperm motility and morphology was assessed prior to IVF. Additionally, only semen from the bulls with proven performance was utilized. Cumulus cells were stripped away from the oocytes 18–22 hours after insemination. The presumed embryos were then cultured for 6 days in synthetic oviductal fluid (SOF) medium as previously described [22]. On day 6 of SOF culture (eight days after OPU), embryos were selected and graded according to the IETS embryo grading system.

### Statistical Analyses

The objective of our statistical analysis was to determine if the reproductive output in cattle clones is significantly different from that of their genetic donors. The individual donor animal was considered as the experimental unit for all analyses, and group sizes are shown in Table 2. For those animals that were used for repeated AI or IVF procedures, the average for each experimental outcome (oocytes, flushed embryos or transferable embryos) was calculated on a per animal basis (*e.g.*, average number of oocytes produced per animal). Mixed model analyses were performed (Mixed procedure with REML estimation method, SAS 9.3) to test for the main effect of breeding group (genetic donor or clone) using the Satterthwaite approximation for degrees of freedom method to account for any apparent difference in variation between the compared groups. A significant effect of treatment group was inferred when *P*<0.05. The average age of the breeding animal over the course of the collection period and the method of semen preparation were included as covariate factors. Because the donor animals, sires and breeds selected for these studies were chosen at random from a broader population of cattle, these parameters were included as random factors in the statistical model. Graphical analysis of conditional Pearson and studentized residual plots revealed that the statistical model satisfied the assumptions of normality and equal variance for this dataset. Separately, the SAS ttest procedure was used to compare variances between genetic donor and clone breeding groups for each parameter of reproductive performance. In most cases, *P* value was >0.05 for the test of equal variance (method: folded F). In order to further examine reproductive performance for each individual procedure as a function of the age of the animal, regression analysis was performed for the first 6 years (overlapping age for genetic donors and clones). Additionally, the median values analysis for each of the reproductive parameters was also performed.

**Table 2:** Reproductive performance of cattle clones and their genetic donors after AI and IVF.

	Superovulation with AI				Ovum pickup with IVF			
Breeding group	*N*	Age	Flushed Embryos	Transferable Embryos	*N*	Age	Oocytes	Transferable Embryos
Genetic donor (Average ± SD)	34	6.65±3.20	10.47±4.75	4.77±3.15	25	9.32±4.04	17.7±8.14	5.09±4.39
Clone (Average ± SD)	85	3.37±1.30	9.68±6.57	4.42±3.38	61	3.90±1.58	18.9±9.00	6.04±5.85
Equal variance test (*P* value)^a^		<0.0001	0.0391	0.6589		<0.0001	0.5963	0.1205
Minimum difference^b^			3.07	1.86			5.67	3.27

*Notes*: Values shown are the number of animals (*N*) per breeding group or the average values for age, number of flushed embryos, number of transferable embryos or number of oocytes ± standard deviation for each breeding group.

^a^ The SAS ttest procedure was used to compare variances between genetic donor and clone breeding groups. Results of the test of equal variances (method: folded F) are shown for each comparison category.

^b^ Retrospective power analyses were performed (*t*-test with two-tailed α = 0.05) to determine the minimum significant difference detectable (at 80% power) for all comparisons between genetic donor and clone shown above (except age). For example, with *N = *34 for donors (σ = 4.75) and *N = *85 for clones (σ = 6.57), we would have 80% power to detect a significant difference of 3.07 flushed embryos generated by superovulation with AI.

## Results and Discussion

This longitudinal study represents the largest data set available for any species on the reproductive performance of female clones and their genetic donors as measured by their embryo production outcomes in a commercial embryo production program. The majority of previously published reports compare clones to their age-matched controls [3], [10]–[13], [15] rather than their genetic donors. Additionally, in previous studies the reproductive performance of clones was typically assessed either after AI or natural mating. Therefore, this large-scale study provides a unique comparison of clones to their genetic donors. Furthermore, to our knowledge, this is the first longitudinal study on the reproductive performance of cattle clones using OPU-IVF procedure. In total, ninety-six clones and their forty corresponding genetic donors were used in these two longitudinal studies. The most important finding of this large-scale longitudinal study is that reproductive performance of cattle produced by SCNT was not different compared to their corresponding genetic donors.

### Longitudinal Study 1

**Figure 1:**
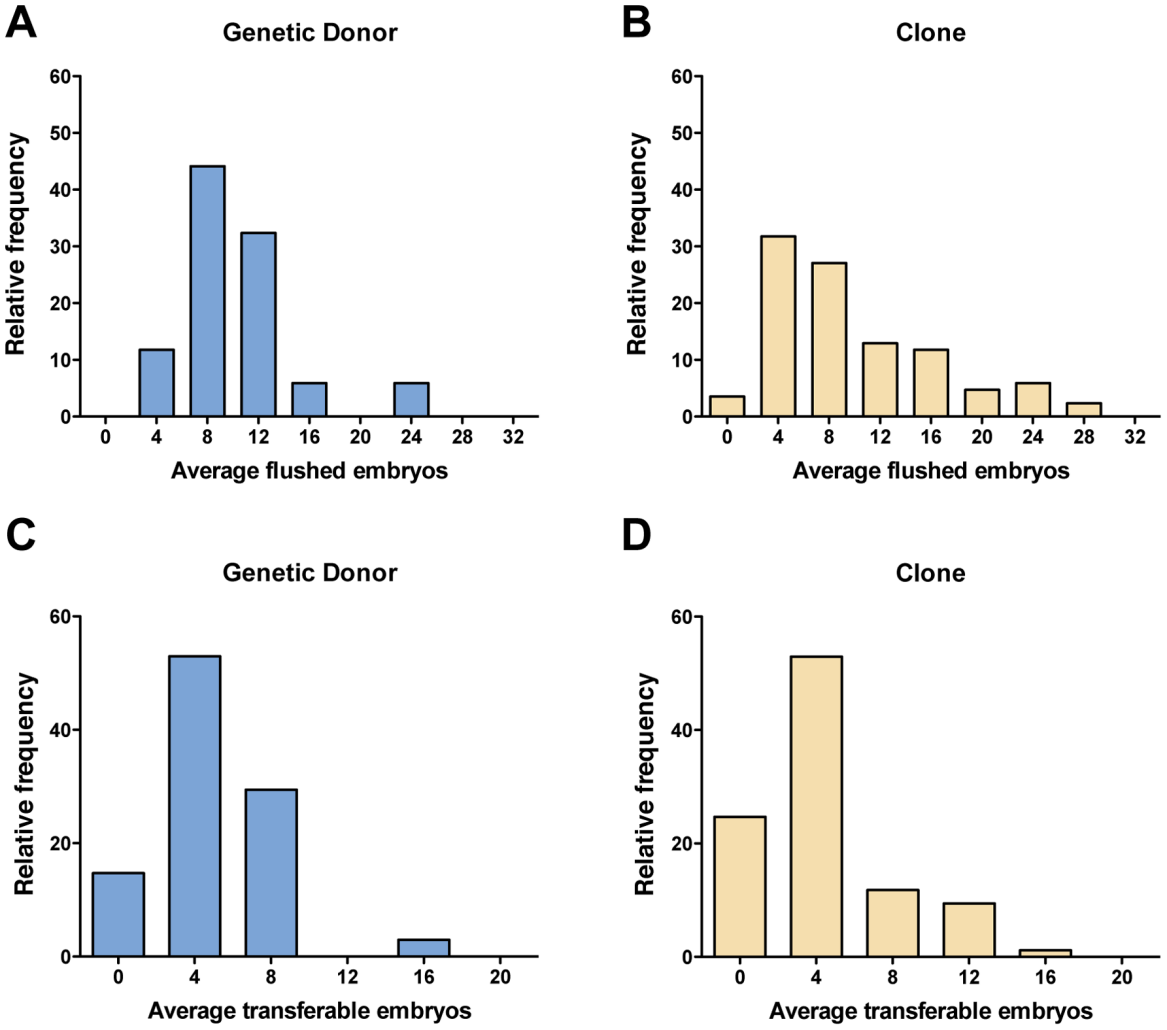
Relative frequency histograms showing the distribution of average values for flushed embryos (A, B) or transferable embryos per animal (C, D) generated by superovulation followed by AI and embryo collection in genetic donors (A, C) or clones (B, D).

**Figure 2:**
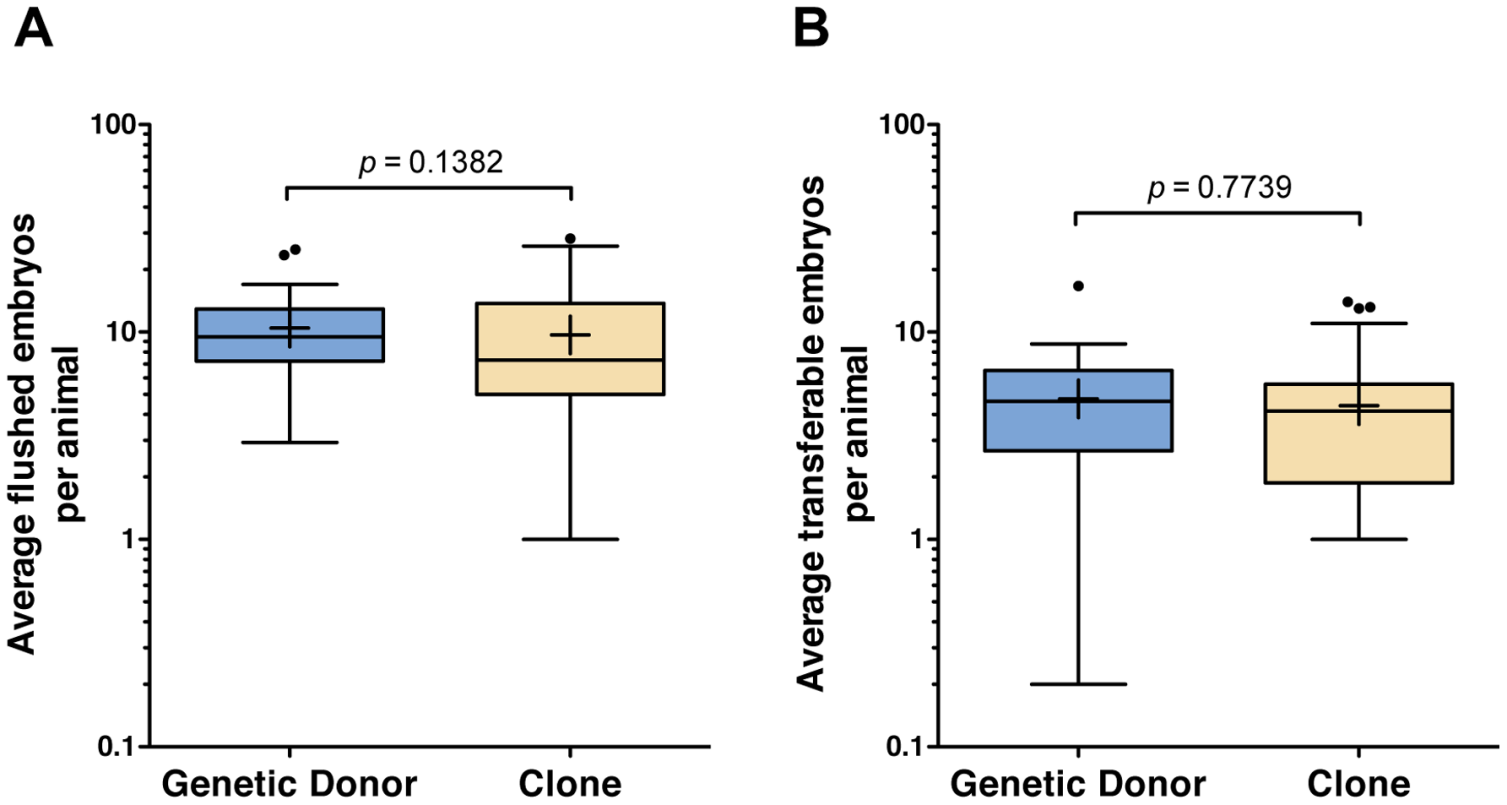
Reproductive performance of clones and their corresponding genetic donors measured by number of flushed embryos and transferable embryos recovered following superovulation, AI and embryo collection. The average number of flushed embryos (A) or transferable embryos (B) per animal are represented as box-and-whisker plots (whiskers are 1.5× the interquartile range and the plus symbol indicates the mean) for each treatment group. *P* values of the type III tests for fixed effects (SAS Mixed procedure) are shown.

Eighty-five cattle clones and their 34 genetic donors were used in this study. Multiple clones were produced from some of the genetic donors (Table 1). Several superovulation, AI and embryo collection procedures were conducted for the majority of the animals. In total, 674 embryo collections were performed (303 collections from clones and 371 from the genetic donors), which resulted in 1,535 and 1,903 transferable embryos, respectively. Figure 1 illustrates the distribution of average flushed embryos or average transferable embryos generated by superovulation followed by AI and embryo collection in genetic donors (A, C) or clones (B, D). As shown in Figure 2, the average number of flushed embryos obtained was not statistically different between the genetic donors and clones (10.47±4.75 and 9.69±6.57, respectively; *P = *0.14); likewise, the number of embryos suitable for transfer was also not statistically different in clones compared to their genetic donors (4.42±3.38 and 4.77±3.15, respectively; *P = *0.77) (Tables 2 and 3). Retrospective power analyses were performed (*t*-test with two-tailed α = 0.05) to determine the minimum significant difference detectable using variance data for each of the reproductive parameters measured. With *N = *34 donors and *N = *85 clones, this test has 80% power to detect a significant difference of 3.07 flushed embryos (σ = 4.75 for donors, 6.57 for clones) or 1.86 transferable embryos (σ = 3.15 for donors, 3.38 for clones) generated by superovulation with AI. Similar statistical observations were made when median values were calculated for flushed or transferable embryos, as shown in Tables S1 and S2.

**Table 3:** Statistical results (*P* values) of mixed models analyses for reproductive performance.

Factors	Superovulation with AI		Ovum pickup with IVF	
	Flushed embryos	Transferable embryos	Oocytes	Transferable embryos
Breeding group	0.1382	0.7739	0.3306	0.9713
Average age	0.5931	0.1204	0.4112	0.9183
Semen preparation method	0.8943	0.7060	N/A	0.5561

*Note:* Using the average values for reproductive performance measures, mixed model analyses were performed (Mixed procedure with REML estimation method, SAS 9.3) to test for the main effects of breeding group (genetic donor or clone). The Satterthwaite approximation for degrees of freedom was used to account for any apparent unequal variance between observational groups. The average age of the embryo donor during the evaluation period and the method of semen preparation were included as covariates, while the breed of the donor, sire identification and embryo donor identification were included as random factors in the model. The statistical analysis for oocytes generated by ovum pickup with IVF did not include semen preparation method as a covariate (N/A, not applicable). Values shown are *P*-values for type 3 test of fixed effects.

The rate of embryo production after AI in clones in this study (average of 4.4 embryos per procedure) was similar to the rate of 4.5 embryos per procedure reported previously [10]. Moreover, Panarace, *et al*. used 21 female clones (heifers and cows) in an embryo transfer program and observed no difference in the embryo production rates between clones and their control counterparts collected at the same farm [10]. Additionally, 74 heifers and cows were bred either naturally or by AI in this study; all of these animals conceived after breeding at one or two estrus periods, indicative of normal breeding rates. The calving rate was also reported to be within a normal range [10]. Although embryo production was the primary endpoint used to assess reproductive performance in clones and their genetic donors, we would also expect that conception and delivery rates in these animals would not differ based on previously published data in multiple species [3], [10], [14], [15].

### Longitudinal Study 2

OPU-IVF has proven to be a highly successful, low-invasive procedure that is often used when there is high demand for offspring from a particular donor cow [24], [25]. Combining this technique with cloning can help to leverage elite maternal genetics even further. Two parameters were used to compare the IVF results of genetic donors and their comparable clones: the number of oocytes aspirated per OPU and the number of transferable embryos produced by IVF. In most cases OPU-IVF was performed multiple times for each genetic donor or clone, and average values for the number of oocytes aspirated or the number of transferable embryos were calculated. FSH stimulation was administered prior to OPU to increase the numbers of follicles and oocytes and to improve blastocyst development rate [26].

**Figure 3:**
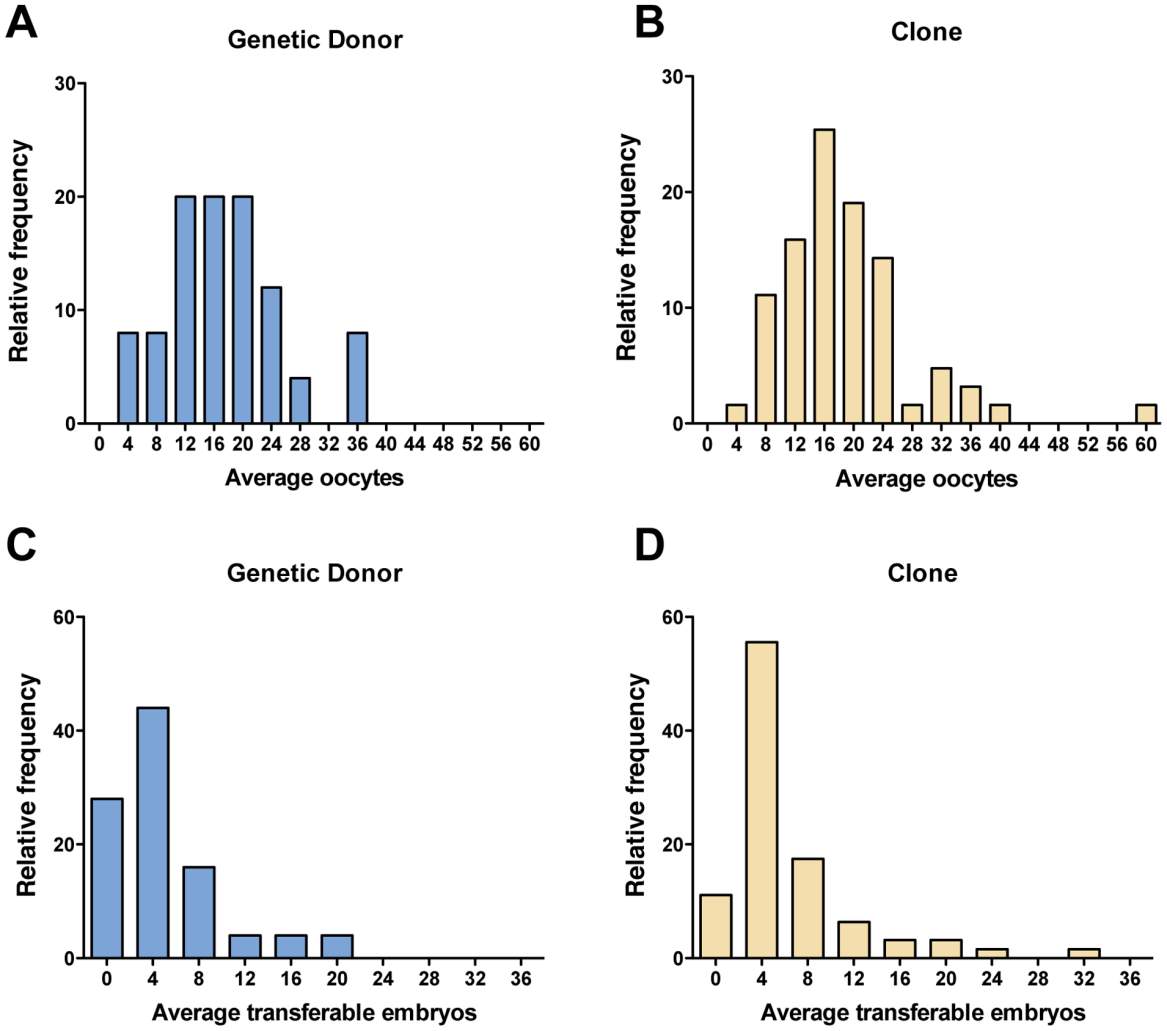
Relative frequency histograms showing the distribution of average values for oocytes (A, B) or transferable embryos (C, D) per animal generated by ovum pick-up followed by IVF in genetic donors (A, C) or clones (B, D).

**Figure 4:**
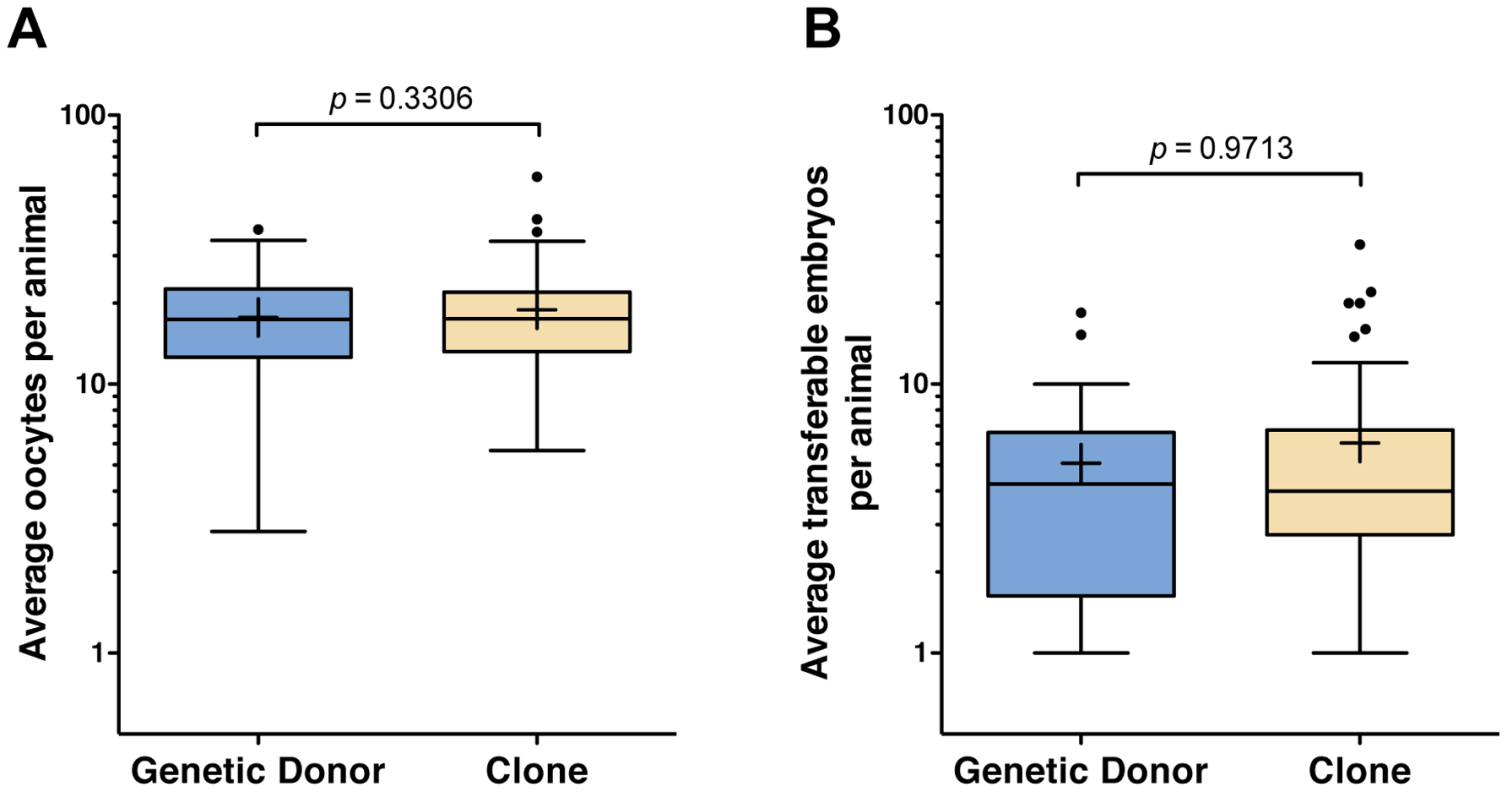
Reproductive performance of clones and their corresponding genetic donors by OPU-IVF procedure. The average number of oocytes (A) or transferable embryos (B) per animal are represented as box-and-whisker plots (whiskers are 1.5× the interquartile range and the plus symbol indicates the mean) for each breeding group. *P* values of the type III tests for fixed effects (SAS Mixed procedure) are shown.

Over the six-year period of evaluation, OPU resulted in the collection of 22,306 oocytes (12,008 from clones and 10,298 from the genetic donors) during 1,251 IVF sessions (655 for clones and 596 for the genetic donors) that ultimately generated 5,775 transferable embryos (3,082 from the clones and 2,693 from the genetic donors). Very similar distributions for the number of oocytes collected and the number of transferable embryos produced by IVF were observed for genetic donors and clones (Figure 3). Similar to our observations in study 1, no significant differences were apparent in the reproductive performance of clones compared to their genetic donors for the number of oocytes retrieved via OPU (*P* = 0.33) or the number of transferable embryos generated by IVF (*P* = 0.97) (Figure 4). Retrospective power analyses were performed (*t*-test with two-tailed α = 0.05) to determine the minimum significant difference detectable using variance data for each of the reproductive parameters measured. With *N = *25 donors and *N = *61 clones, this test has 80% power to detect a significant difference of 5.67 oocytes (σ = 8.14 for donors, 9.00 for clones) or 3.27 transferable embryos (σ = 4.39 for donors, 5.85 for clones) generated by superovulation with AI. Similar statistical observations were made when a median was calculated for either collected oocytes or transferable embryos, as shown in Tables S1 and S2.

**Figure 5:**
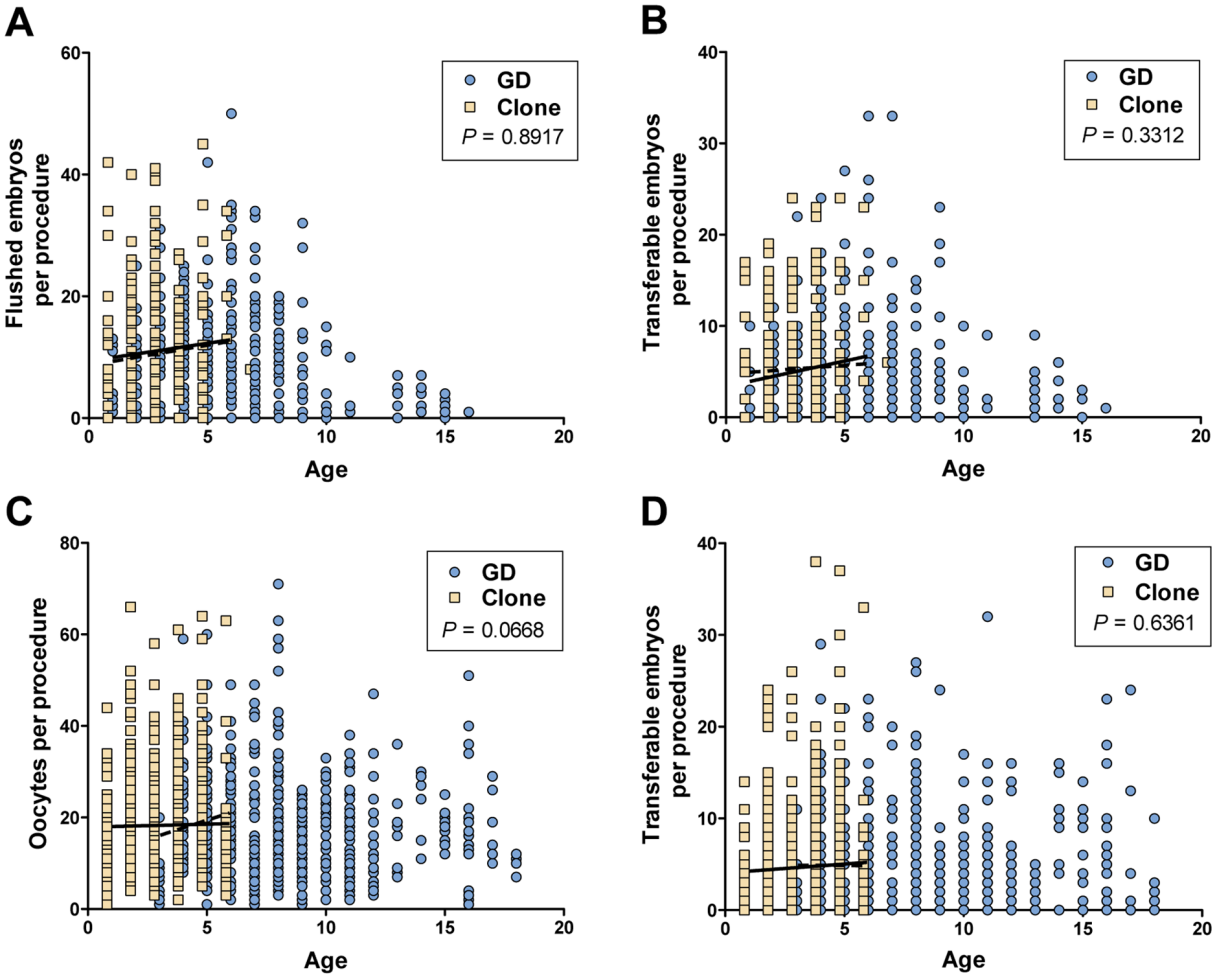
Reproductive performance of clones and corresponding genetic donors (GD) as a function of age. Data for each individual procedure are shown according to the age of the animal at the time of the procedure as follows: (A) the numbers of flushed embryos or (B) transferable embryos generated by superovulation followed by AI and embryo collection, and (C) the numbers of oocytes or (D) transferable embryos obtained by OPU-IVF. All ages were recorded as years; data for clones are left shifted so that symbols for genetic donors are visible. Lines are the linear regressions for genetic donors (dashed) or clone (solid) groups for animals aged 1 to 6 years. *P* values for ANCOVA of the slopes of regression lines (GraphPad Prism) are shown.

For both longitudinal studies, reproductive performance was not significantly affected by either the method of semen preparation or the average age of the animal (Table 3). The effect of age of donor cows on oocyte and embryo production has been thoroughly investigated over the last 50 years [24], [27], [28]. A curvilinear effect of the age of donor on the number of oocytes and embryos collected was reported between 2 and 14 years of age with the maximum response between 6 and 7 years of age [28]. However, the number and percent of transferable embryos is the same for ages 3 to 6 and 7 to 9 years, but the results for ages 3 to 9 are greater than for 10 to 22 year old group [27]. Embryo collections outcomes are also typically lower for female cattle under 3 years of age [28]. Considering that the GDs were older than the clones especially in OPU-IVF study, we further examined reproductive performance for each individual procedure as a function of the age of the animal at the time of procedure (Figure 5). Generally, reproductive performance with respect to the age of the animal was at least as high in cloned animals as in their genetic donors for animals 1 to 6 years of age (overlapping age for GDs and clones); slopes of regression lines were not significantly different between genetic donor and clone groups for flushed and transferable embryos generated by AI followed by embryo collection (Figure 5A, B) or for oocytes and transferable embryos generated by OPU-IVF (Figure 5C, D) (*P*>0.05 by ANCOVA for slope of regression lines, GraphPad Prism v.5). Although oocyte production by genetic donors appears positively correlated with animal age (Figure 5C), this correlation is not significantly different from that of clones (*P = *0.067). More importantly, the most commercially relevant outcome (the number of transferable embryos) is highly similar (*P = *0.64) for these two groups when matched by age (Figure 5D). The oldest clones used in this study were 6–7 years of age. Our group continues to evaluate this cohort of animals to determine the impact of increasing age on reproductive performance in clones.

Various aspects of reproductive performance in cattle produced by SCNT were previously investigated since demonstration of normal reproductive capability is critical for the technology utilization. For example, changes in plasma progesterone during the pre- and post-pubertal periods were reported to be similar in clones compared to the non-cloned cows [5]. Additionally, estrous cycle length, ovulatory follicular diameter, number of follicular waves and hormonal profiles (LH, FSH, estradiol, and progesterone) were observed to be similar in clones and control heifers [11]. However, this group also reported that cloned heifers reached puberty later than control animals matched for age and weight, although these controls were not of the genetic donors for the clones. The specific genetic background of clones could have been a determining factor of the puberty onset in these animals, yet no puberty records were available for the clones’ genetic donors [11]. Furthermore, offspring of clones have normal karyotypes and age appropriate telomere lengths; also, growth and reproductive profiles do not differ from age- and breed-matched controls [3]. Ortegon, *et al.* reported that the female progeny of a cloned bull (Starbuck II) reached puberty at the expected age (10–12 months) and weight (318–365 kg) [3]. In our study, onset of puberty was not evaluated, as the animals were not raised at the Trans Ova Genetics facilities. However, we observed that long-term reproductive performance of clones was comparable to their corresponding genetic donors. Furthermore, our overall embryo production rates are comparable with the current data reported by other investigators [25].

In summary, the results of these large-scale longitudinal studies, including nearly 2,000 ART procedures and more than 9,200 transferable embryos produced, convincingly demonstrate that the reproductive performance of clones is comparable to their genetic donors for the production of transferable embryos after either superovulation and AI followed by embryo collection or OPU-IVF. Therefore, the cloning technology can be effectively used for propagation of highly desirable genetic lines. Cloning is the only reproductive method enabling breeders to multiply the best female animals and increase the impact of their genetics. This study is the largest to be reported on the reproductive performance of cloned cattle showing that cloning technology combined with superovulation, AI and embryo collection or OPU-IVF provides a valuable tool for faster dissemination of superior maternal genetics. Also, these data further support previous reports that the reproductive capabilities of cloned cattle are equal to that of cattle produced by conventional methods.

## Supporting Information

Table S1 (DOCX)

Table S2 (DOCX)
