# Supplementary material for: Longitudinal Study of Reproductive Performance of Female Cattle Produced by Somatic Cell Nuclear Transfer
Source: PLoS One. 2013 Dec 31;8(12):e84283. doi: 10.1371/journal.pone.0084283 (PMC3877258; doi:10.1371/journal.pone.0084283)
Supplement: Table S1 — (DOCX) [file pone.0084283.s001.docx]

| **Table S1. Reproductive performance of cattle clones and their genetic donors after AI and IVF** | | | | | | | | | |
| --- | --- | --- | --- | --- | --- | --- | --- | --- | --- |
|  | **Superovulation with AI** | | | |  | **Ovum pickup with IVF** | | | |
| **Breeding group** | ***N*** | **Age** | **Flushed Embryos** | **Transferable Embryos** |  | ***N*** | **Age** | **Oocytes** | **Transferable Embryos** |
| Genetic donor  (Median ± SD) | 34 | 6.65 ± 3.20 | 10.0 ± 5.23 | 3.60 ± 3.15 |  | 25 | 9.32 ± 4.04 | 17.0 ± 7.92 | 4.22 ± 4.96 |
| Clone  (Median ± SD) | 85 | 3.37 ± 1.30 | 9.25 ± 6.36 | 4.05 ± 3.81 |  | 61 | 3.90 ± 1.58 | 18.3 ± 8.96 | 5.40 ± 4.94 |
| Equal variance test  (*P* value) ^a^ |  |  | 0.2092 | 0.2191 |  |  |  | 0.5075 | 0.3274 |
| Minimum difference ^b^ |  |  | 3.21 | 1.94 |  |  |  | 5.60 | 3.36 |

*Notes*: Values shown are the number of animals (*N*) per breeding group or the median values for age, number of flushed embryos, number of transferable embryos or number of oocytes ± standard deviation for each breeding group.

^a^ The SAS ttest procedure was used to compare variances between genetic donor and clone breeding groups. Results of the test of equal variances (method: folded F) are shown for each comparison category.

^b^ Retrospective power analyses were performed (*t*-test with two-tailed α=0.05) to determine the minimum significant difference detectable (at 80% power) for all comparisons between genetic donor and clone shown above (except age). For example, with *N* = 34 for donors (σ=5.23) and *N*=85 for clones (σ=6.36), we would have 80% power to detect a significant difference of 3.21 flushed embryos generated by superovulation with AI.
