# Supplementary material for: Longitudinal Study of Reproductive Performance of Female Cattle Produced by Somatic Cell Nuclear Transfer
Source: PLoS One. 2013 Dec 31;8(12):e84283. doi: 10.1371/journal.pone.0084283 (PMC3877258; doi:10.1371/journal.pone.0084283)
Supplement: Table S2 — (DOCX) [file pone.0084283.s002.docx]

| **Table S2. Statistical results (*P* values) of mixed models analyses for reproductive performance**. | | | | | |
| --- | --- | --- | --- | --- | --- |
| **Factors** | **Superovulation with AI** | |  | **Ovum pickup with IVF** | |
|  | **Flushed embryos** | **Transferable embryos** |  | **Oocytes** | **Transferable embryos** |
| Breeding group | 0.8320 | 0.6194 |  | 0.1401 | 0.2064 |
| Average age | 0.014 | 0.4598 |  | 0.5918 | 0.9503 |
| Semen preparation method | 0.3615 | 0.8375 |  | N/A | 0.4211 |

*Note:* Using the median values for reproductive performance measures, mixed model analyses were performed (Mixed procedure with REML estimation method, SAS 9.3) to test for the main effects of breeding group (genetic donor or clone). The Satterthwaite approximation for degrees of freedom was used to account for any apparent unequal variance between observational groups. The average age of the donor during the evaluation period and the method of semen preparation were included as covariates, while the breed of the donor, sire identification and donor identification were included as random factors in the model. The statistical analysis for oocytes generated by ovum pickup with IVF did not include semen preparation method as a covariate (N/A, not applicable). Values shown are *P*-values for type 3 test of fixed effects.
